# Process Evaluation of an eHealth Intervention Implemented into General Practice: General Practitioners’ and Patients’ Views

**DOI:** 10.3390/ijerph15071475

**Published:** 2018-07-12

**Authors:** Louise Poppe, Jolien Plaete, Nele Huys, Maïté Verloigne, Myriam Deveugele, Ilse De Bourdeaudhuij, Geert Crombez

**Affiliations:** 1 Department of Movement and Sports Sciences, Ghent University, 9000 Gent, Belgium; jolien.plaete@gezondleven.be (J.P.); nele.huys@ugent.be (N.H.); maite.verloigne@ugent.be (M.V.); ilse.debourdeaudhuij@ugent.be (I.D.B.); 2 Department of Experimental Clinical and Health Psychology, Ghent University, 9000 Gent, Belgium; geert.crombez@ugent.be; 3 Department of General Practice and Primary Health Care, Ghent University, 9000 Gent, Belgium; myriam.deveugele@ugent.be

**Keywords:** health promotion, eHealth, general practice, self-regulation, physical activity, healthy nutrition

## Abstract

(1) Background: It has been shown that online interventions can be enhanced by providing additional support; accordingly, we developed an implementation plan for the use of an eHealth intervention targeting physical activity and healthy nutrition in collaboration with general practitioners (GPs). In this study, GPs and patients evaluated the actual implementation; (2) Methods: Two hundred and thirty two patients completed the feasibility questionnaire regarding the implementation of “MyPlan 1.0” in general practice. Individual interviews were conducted with 15 GPs who implemented “MyPlan 1.0” into their daily work flow; (3) Results: The majority of the patients indicated that general practice was an appropriate setting to implement the online intervention. However, patients were not personally addressed by GPs and advice/action plans were not discussed with the GPs. The GPs indicated that this problem was caused by the severe time restrictions in general practice. GPs also seemed to select those patients who they believed to be able to use (e.g., highly educated patients) and to benefit from the intervention (e.g., patients with overweight); (4) Conclusions: Although GPs were involved in the development of the online intervention and its implementation plan, the programme was not used in general practice as intended.

## 1. Introduction

The promotion of physical activity (PA) and a healthy diet plays a key role in the prevention of non-communicable diseases [1]. General practitioners (GPs) have a significant role in disease prevention and health promotion [2,3,4]. Approximately 77% of adults consult their GP once a year [5], and GPs are considered to be a credible source for health promotion [6,7]. Although GPs indicate that health promotion is important, its implementation in general practice remains limited due to barriers such as lack of time and training as well as other priorities in patient care [2,7,8,9,10,11,12].

eHealth refers to health services or information provided via the Internet and related technologies [13]. eHealth interventions have shown to be effective in promoting physical activity and a healthy diet [14]. They often provide individual feedback on health behaviours by applying algorithms to participants’ answers to an online questionnaire [15]. Their effectivity may be further improved by integrating self-regulation techniques (e.g., personal goal setting, coping, and action planning) [14,16]. In so doing, computer-tailored interventions may assume some of the tasks of GPs, prompting them to address health promotion during consultation [12,17]. In addition, the reach and use of computer-tailored interventions can be enhanced by the provision of additional support [18]. Thus, GPs may increase the effects of eHealth interventions by providing support and discussing the computer-tailored advice of patients [16,19,20].

Taking these findings into account, we developed “MyPlan 1.0”, a computer-tailored eHealth intervention based on self-regulation. “MyPlan 1.0” targets PA as well as fruit and vegetable intake of adults visiting general practice. The Belgian organisation of general practice differs in some aspects from other European countries. In Belgium, there is an “open market” system, which allows patients to consult more than one GP. Typically, a consultation with the GP lasts about fifteen minutes, which is comparable to consultations in Switzerland but longer than consultations in Germany or Spain [21]. Furthermore, in contrast with, e.g., GPs in the Netherlands, GPs in Belgium have a stronger disease-centred focus [22]. To optimally adapt the online intervention, GPs were consulted in the creation of “MyPlan 1.0” via focus groups [12], and many of their proposals were taken into account: making use of tablet computers to deliver the intervention, providing different choice options for GPs to introduce the intervention to patients, providing the opportunity for patients to halt and resume the programme, providing a security system to use the tablet in the waiting room, providing attractive posters in the waiting room, providing the opportunity to patients to discuss their tailored feedback and action plans with their GP, and the use of online follow-up modules. “MyPlan 1.0” has been shown to be effective in increasing levels of PA as well as fruit and vegetable intake but has high levels of attrition [23,24].

The aim of this study was to investigate how patients and GPs experienced the implementation of “MyPlan 1.0” into general practice. Such process evaluation may help us to understand the reasons underlying the attrition and to formulate recommendations for the future dissemination of similar interventions.

## 2. Materials and Methods

### 2.1. “MyPlan 1.0”

“MyPlan 1.0” is an online programme which is based on self-regulation theory and supports participants in being more physically active as well as in consuming more fruit and/or vegetables. The programme consists of three sessions. In the first session, users complete a questionnaire assessing demographic information (e.g., age, gender, etc.), decide which behaviour they will focus on, and fill out a questionnaire measuring their current level of the selected behaviour. On the basis of their input, personal advice is given. Users then create a specific plan for action and decide how they will tackle potential barriers. One week later, users receive an e-mail, inviting them to go through the second session. During this session, users evaluate their change, are offered the possibility to adapt their plan, and are prompted to provide solutions for potential barriers. Finally, after one month, users are invited for the third session, which has the same structure as the second session. The content of “MyPlan 1.0” is described in detail elsewhere [25].

### 2.2. Implementation of “MyPlan 1.0” in General Practice

**Figure 1:**
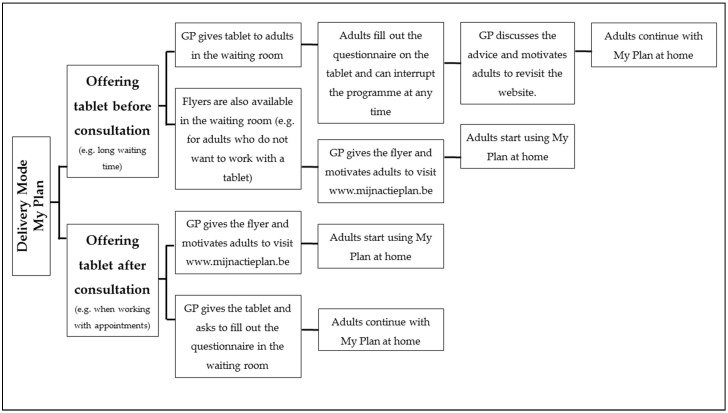
Flow-chart with the delivery options for the general practitioners (GPs).

“MyPlan 1.0” was implemented in 19 general practices in Flanders. The practices were a convenience sample recruited via email messages, telephone calls, and advertisements on association websites of GPs. Both the researchers and the GPs recruited patients (adults ≥ 18 years) in the general practices. The recruitment of a patient took approximately three minutes. Various options for delivery were provided, and GPs selected the delivery mode that best fit in their working system. The options were presented to the GPs by means of a flow-chart (see Figure 1). Patients were informed that they could discuss their advice or action plan generated by the programme with their GP in their next consultation.

### 2.3. Assessment of Patients’ Views Regarding the Implementation of “MyPlan 1.0”

After the first module, patients completed an evaluation questionnaire regarding the implementation of the intervention. There were questions about the recruitment method, feasibility of implementing the intervention in general practice, and discussion of the action plan/advice with the GP. The questionnaire was specifically designed for the purpose of this study. It was iteratively developed by experts in health promotion and health psychology to ensure that all important aspects of the process evaluation were covered (content validity).

### 2.4. Assessment of GPs’ Views Regarding the Implementation of “MyPlan 1.0”

At the end of the study, GPs were invited via phone to participate in an individual interview on the implementation of “MyPlan 1.0” in their practice. The interview guide consisted of open-ended questions and addressed five topics: (1) the delivery mode (i.e., use of the tablet and flyers); (2) the target group (i.e., was the intervention delivered to healthy patients and thus used for primary prevention); (3) barriers and facilitating factors for implementation; (4) discussion of the advice and action plan by GPs and (5) other ideas for future implementation. Interviews were led by two interviewers and lasted, on average, 30 min.

### 2.5. Ethical Approval

Ethical approval (approval number: 670201319313) was provided by the Ghent University Ethics Committee, and participants provided a written informed consent.

### 2.6. Sample

#### 2.6.1. Patients

**Figure 2:**
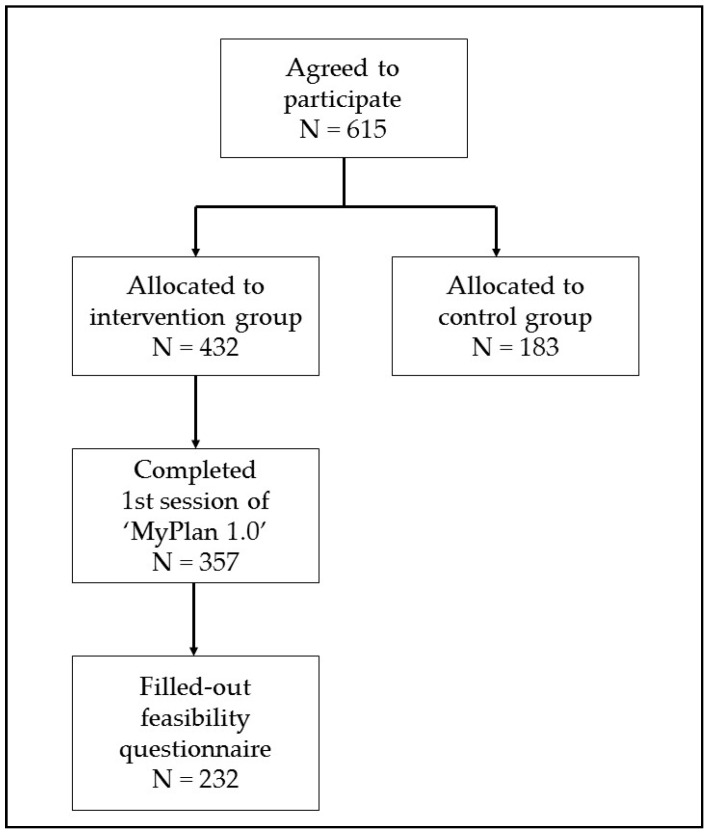
Flow of the participants.

Figure 2 shows the flow of the participants. Two hundred and thirty two patients of the 357 patients (response rate = 65%) who completed the first intervention module filled out the feasibility questionnaire. For these patients, the average number of GP visits per year was 4.80 (±3.82), mean age was 43.5 (±14.0) years, and mean Body Mass Index (BMI) was 25.5 (±5.1) kg/m^2^. In total, 50.5% of these patients were highly educated (i.e., patients with a university or college degree).

#### 2.6.2. GPs

Fifteen of the 19 GPs volunteered for the interviews. The mean age was 47.2 (±12.2) years and the mean number of years of experience was 21.7 (±12.8) years. Eight (53%) were male, nine GPs (60%) worked in group practices, and six worked alone (40%). A practice assistant was employed by five GPs (33%). Three GPs (20%) had a computer in the waiting room and 12 GPs (80%) had wireless internet in their practice.

### 2.7. Data Analysis

Descriptive statistics using SPSS (IBM Corporation, Somers, NY, USA) were used to describe the patients’ questionnaire data. The interview data were thematically analysed via NVivo software (Version 11, QSR International Pty. Ltd., Melbourne, Australia, 2015) in several phases [26]. First, a coding scheme was developed, which was based on both the focus group study with the GPs [12] and other studies on the delivery of health promotion interventions in general practice [2,3,7,8,27,28]. This scheme consisted of the five themes addressed during the interview. Second, two researchers (N.H. and J.P.) independently started coding, using a combination of axial coding and inductive coding. Accordingly, other sub-themes that arose in the transcripts were added to form the final coding template [26,29]. Disagreements were discussed by the two researchers until consensus was reached and interrater reliability was good (single measures ICC = 0.72). The final coding scheme was used to code all transcripts. Finally, all codes of the transcripts were mutually compared and interpreted [26,29,30]. The completed Consolidated criteria for Reporting Qualitative research (COREQ) checklist is provided in the Supplementary File.

## 3. Results

### 3.1. The View of the Patients

Of the 232 participants who filled out the feasibility questionnaire, 72 (31%) indicated that they started the intervention on a tablet in general practice after a researcher handed out the tablet, and 5 (2.2%) after the GP gave them the tablet. Of the patients who answered that they started the intervention at home after having received a flyer, 89 (38.4%) indicated that they received the flyer from a researcher, and 37 (16%) received it from a GP. Seventeen (7.3%) participants who completed the feasibility questionnaire indicated that they found the flyer in the waiting room without any personal contact. Twelve (5.2%) participants did not answer this question.

Using a flyer to deliver the intervention was indicated as the best option by 136 (58.6%) participants and the use of the tablet by 96 (41.3%) participants. Most participants (*n* = 167; 72%) indicated general practice as a feasible setting to implement “MyPlan 1.0”, 60 (26%) indicated had no opinion, and 5 (2%) indicated general practice as a non-feasible setting. The most endorsed reasons for considering the general practice as a feasible setting are provided in Table 1. The majority of participants (*n* = 170; 73.3%) indicated they did not discuss the intervention with their GP. When participants did, the following issues were addressed with their GP: reasons and invitation to participate (70.1%) as well as an explanation about the content of the intervention (29.9%). None of the participants indicated they discussed their personal advice or action plan with their GP.

### 3.2. The View of the GPs

#### 3.2.1. The Delivery Mode

Only a minority of the GPs indicated to use the tablet in their practice. Reasons not to install the tablet were risk of theft, a weak internet signal, recharging problems, inexperience in tablet use or lack of knowledge to solve technical problems. GPs also indicated reasons why patients did not use the tablet, such as patients were not familiar with the use of a tablet, the purpose not clear enough for patients, patients were not willing to spent time to use the tablet, and the variation in time spent in the waiting room or during consultation. Some GPs stated a belief in the use of eHealth interventions for future health promotion in general practice because of the upcoming use of technology. Others indicated it was too soon to integrate eHealth interventions into general practice.

“Because of the profile of our patients (low socio-economic status), we thought the tablet might not be handled with care. That’s the reason why we did not use the tablet.”

(Female, 12 years of experience, group practice)

“I think we are not ready yet to use tablets”

(Male, 35 years of experience, group practice)

In comparison, flyers were used by all GPs. Most GPs personally gave the flyers to patients during or after consultation and provided some extra information about the eHealth programme. In some practices, the flyers were handed out by the practice assistance or put in the waiting room without personal contact. GPs indicated that most patients were interested when they received the flyer; GPs also expected higher participation rates when personally addressing patients. Flyers seemed easier to use than tablets, but some GPs noticed that solely handing out flyers might not motivate patients enough to use the eHealth programme.

“You cannot solely give a flyer to people you should motivate, because then I see it on their faces: they will throw it in the paper bin.”

(Female, 12 years of experience, group practice)

Posters regarding “MyPlan 1.0” were put on a visible place in each waiting room of the general practices. GPs mentioned that the posters evoked some questions about the project but doubted their effect on patients’ participation in the intervention.

“There are already a lot of posters in our waiting room. I doubt that people will spontaneously participate in the study by just seeing the poster.”

(Female, 5 years of experience, group practice)

#### 3.2.2. The Target Group

Some GPs stated that they approached all patients as indicated in the study protocol, whereas others only approached patients with health conditions related to an unhealthy diet or physical inactivity (e.g., high BMI) or patients who came for a blood sample, weight control, or check-up.

“It depends on the situation. But to some of my patients I had to give the advice to eat more healthy and be more physically active. Now there was the possibility to do it via that application and I could bring it up this way.”

(Male, 29 years of experience, group practice)

Most GPs mentioned that they mainly recruited middle-aged patients. A GP cited that this was because older patients are less keen to work with tablets, and young patients, especially young mothers, are too busy to participate. One GP mentioned that participants from all ages were willing to participate.

“I selected my patients based on whether I knew they could work with a computer. I could for example not ask persons of 80 years or older, for whom I know cannot work with or do not have a computer, to participate in this study. However, I did not use any other selection criteria.”

(Male, 10 years of experience, group practice)

According to some GPs, the eHealth programme was too difficult and, therefore, was only suited for patients with a high socio-economic status. GPs also indicated that the programme was difficult to use for patients with a different cultural background or another native language. Furthermore, GPs noticed that patients with a healthy lifestyle were more likely to participate.

“We believe in the power of the eHealth programme when it is used in the right population groups, especially the younger, higher educated patients.”

(Female, 2 years of experience, group practice)

#### 3.2.3. Discussion of the Advice and Action Plan with the GP

GPs stated that few patients discussed the programme with them. When discussing the programme, patients only talked about the experienced difficulties but never mentioned their advice or action plan. GPs mentioned that it should be more emphasized that patients can discuss their advice/action plan with their GP.

“Yesterday, a woman said to me: ‘you told me it would not take long to complete it, but it did take long!’”

(Male, 10 years of experience, group practice)

#### 3.2.4. Barriers for Implementation

The most commonly mentioned problem to use the eHealth programme in general practice was lack of time. GPs highlighted the difficulty to integrate additional tasks into their daily workflow due to an overload of medical and administrative tasks. Furthermore, it was mentioned that the intervention sometimes required an additional consultation to motivate patients. Some GPs mentioned that it took too long for patients to fill out the questionnaire in the waiting room or during consultation.

“I think the programme is too extensive to let patients use it in the practice or in the waiting room”

(Male, 10 years of experience, group practice)

Another reported barrier was patients’ reluctance to participate. According to GPs, the main reasons patients refused participation included the following: too ill, not enough time, not motivated, no interest, too much work, did not see any added value of the intervention, or not ready for it.

“When people are here with the flu, you cannot mention their weight or the eHealth programme. When they are too sick, you just can’t start talking about the eHealth programme.”

(Female, 12 years of experience, group practice)

Another indicated barrier was the necessity of using computer devices. GPs mentioned that some patients did not have a computer and/or sufficient knowledge about it to use it for an eHealth intervention.

“Apparently some of my patients found it too difficult to work with the computer, or had insufficient knowledge about a computer to use the eHealth intervention.”

(Female, 30 years of experience, solo practice)

The period in which the study was conducted (winter, October–February) was considered a difficult period for preventive actions. GPs reported that many patients had the flu and stated that spring or summer would be better periods to implement a health promotion intervention. Other barriers that were mentioned included forgetting to mention the programme or a lack of staff.

“It was difficult because it was a busy period with many patients having the flu. Therefore, there was not enough time to take anything extra.”

(Male, 29 years of experience, solo practice)

#### 3.2.5. Facilitating Factors for Implementation

At the start of the study, a researcher took over the GP’s task to recruit and motivate participants to use “MyPlan 1.0”. GPs were very positive about this and emphasized the need of additional staff (e.g., practice assistant) to take over preventive tasks.

“It is easier because we have a practice assistant, she can offer the eHealth programme to the patients. GPs who don’t have a practice assistant have to do it themselves.”

(Female, 9 years of experience, group practice)

GPs were positive about the tailored advice that was automatically provided by the eHealth programme because they did not have to ask health-related questions or provide advice about health themselves.

“I found this a very interesting project. Especially because the researchers did all the preparatory work and gave us a ‘ready-to-use package’ and programme that provided all the tailored feedback. I really liked it.”

(Male, 38 years of experience, group practice)

GPs appreciated the flow chart that included different options to deliver the intervention. Furthermore, the telephone calls to remind GPs about the study were positively evaluated.

“You should indeed be able to adapt the delivery mode to your own style, because indeed, GPs work in different ways.”

(Male, 29 years of experience, group practice)

#### 3.2.6. New Ideas for Future Implementation

The most important suggestion was the integration of “MyPlan 1.0” in existing medical programmes of GPs. GPs indicated that such reports would be useful as a way to know which patients used the intervention and to see their progress. Some GPs also suggested a link between the intervention and the Global Medical File, a screening questionnaire to detect patients at risk for chronic diseases, because it would be easier to introduce “MyPlan 1.0” after using this questionnaire.

“It would be a good idea to combine the programme with the GMD+ questionnaire, filling in the Global Medical File immediately offers a reason to talk about the programme.”

(Female, 30 years of experience, solo practice)

GPs also gave suggestions to introduce the intervention in a more attractive way, such as installing a bike to conduct an exercise test in the waiting room. Two GPs suggested to integrate “MyPlan 1.0” into a smartphone application that people could download and use on their own mobile phone.

“I think it would be a good idea to use an app for it. Patients will rather use the eHealth programme on their own smartphone. It may be a better idea than working on the tablet. When using an app also more people can use the eHealth programme at the same time.”

(Female, 25 years of experience, solo practice)

Furthermore, GPs suggested that the programme should be implemented in different settings, such as at pharmacies or by dietitians. Some GPs mentioned the reimbursement of separate consultations for preventive health care, in which eHealth programmes like “MyPlan 1.0” can be used.

“Maybe, the programme should not only be spread via us but via different channels, people should also see it in the media. Delivering this programme in pharmacies would be a nice idea, or dietitians could perhaps also use it.”

(Female, 12 years of experience, group practice)

**Figure 3:**
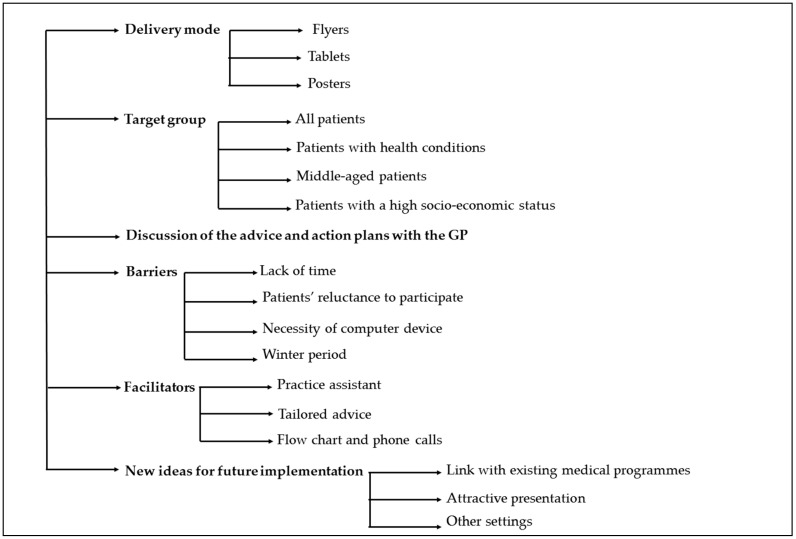
Overview of the themes and subthemes discussed with the GPs.

Figure 3 provides an overview of the themes and subthemes discussed with the GPs.

## 4. Discussion

This study evaluated the process of implementing an eHealth intervention in general practice by assessing the experiences of both patients and GPs. Patients indicated general practice as an appropriate setting to implement “MyPlan 1.0”. Notwithstanding, GPs stated that a lack of time hindered them from fully integrating the programme in their daily workflow.

Lack of time had been mentioned as an important barrier in the prior focus groups with GPs [12]. It was hypothesized that by using an automated, computerized intervention, offering different options to deliver “MyPlan 1.0”, and giving patients the possibility to halt and resume the programme would solve this problem. The current study shows that GPs liked the ready-to-use package. Nevertheless, they still experienced a lack of time to introduce the programme to their patients. This, together with the finding that Belgian GPs are rather disease-centred [22], might explain why they mostly targeted “at risk” patients (e.g., patients with high BMI) and preferred a research assistant to take over this task. In previous studies, it was also found that GPs consider primary prevention to be important, but many of them only apply secondary prevention [9,31].

This raises the question whether eHealth should be encouraged in general practice. None of the patients who used “MyPlan 1.0” discussed their advice or plans with their GP (see also Carroll et al. [32]). This was also the case for those patients who indicated that general practice was an appropriate setting to implement an online intervention. Frequently, these patients mentioned the high reach of varied patients, the available time while waiting, the proper context to discuss a healthy lifestyle, and the trust that patients have in their GP. At first sight, this is disappointing. Nevertheless, we need to take into account that our questionnaire was only completed by patients who finished the first session of the programme. It may be that these patients were already motivated to change their behaviour and thus needed no further encouragement by their GP. Consequently, it may be important to train the next generation of GPs in the use of motivational techniques [33] to address those patients who do not yet have an intention to change.

One limitation to the results of our study is the small sample of (motivated) GPs. Some GPs had already participated in focus groups on this topic and had indicated interest in health promotion; this suggests that the findings cannot be generalised to the entire GP population. Further, it is remarkable that even our motivated sample of GPs was reluctant to discuss patients’ advices and focused on patients with specific health conditions. These findings might serve as a warning to other researchers who create online interventions in the context of general practice. Second, the patients who filled out the questionnaires were those patients who completed the first intervention module. Accordingly, the views of patients who did not participate in the study or who did not finish the first module were not assessed. We also did not record how many patients were approached or how many flyers were taken. Consequently, there is no information available on the number of potentially interested patients. Third, no specific theoretical framework was used to conduct the process evaluation (e.g., Glasgow et al. [34] or Saunders et al. [35]). Finally, our study period (winter) was indicated by GPs as a less feasible period to conduct health promotion. However, in the focus group interviews [12], GPs indicated winter as an appropriate period because many patients visit general practice during this time.

This study revealed some practical considerations when implementing eHealth interventions in general practice. First, patients should be able to complete one session of the programme within the time they spend waiting. If not, the possibility to halt and resume the programme should be highlighted. Second, more expensive and complex tools, such as tablets, should not replace simple tools such as flyers. GPs especially favoured the flyers as tablets could be stolen, damaged, or needed to be charged. In line with these findings, a mobile application might be convenient in which patients would be able to download the programme on their own smartphone. Finally, GPs were more inclined to address patients whom they thought would be able to use “MyPlan 1.0” (i.e., middle-aged adults with a high socio-economic status). However, a previous study of “MyPlan 1.0” indicated that participants of an older age or with a low educational status did not experience the intervention as less acceptable or feasible [36]. This previous study was conducted in a convenience sample during the development of the programme. This is in contrast with the current study which focused on the implementation of the final programme in general practice. Our drop-out study, which was based on the user data of the final programme, confirmed these findings: the level of education did not predict whether a participant finished the first module nor the complete intervention [37]. These results are in line with other research, showing the positive evaluation of computer-tailored programmes by participants with a low educational level [38]. However, whether the intervention effects of “MyPlan 1.0” differ according to educational level needs to be further examined. Also, the use of internet and electronic devices has increased significantly during the past years, suggesting that, in time, a higher number of older people will be able to use eHealth programmes [39]. A broad variety of patients visit general practice, making this setting the ideal opportunity to address various target groups. Our results indicate that stimulating GPs to address a wide variety of patients is important when implementing eHealth in general practice.

## 5. Conclusions

To conclude, this study explored how patients and GPs experienced the implementation of an eHealth intervention in general practice. Patients considered general practice as a good setting to implement “MyPlan 1.0”. GPs enjoyed the automated intervention but still experienced a lack of time to introduce the programme to their patients.

## Figures and Tables

**Table 1:** Patients’ reasons why general practice is a feasible setting to implement “MyPlan 1.0”.

Reasons Why General Practice Is a Feasible Setting to Implement “MyPlan 1.0” According to Patients	*n* = 114
High reach of varied patients	24 (21.1%)
Because you have time when you are waiting in the waiting room	21 (18.4%)
Right context to talk about a healthy diet and PA	21 (18.4%)
GPs’ authority and trust in GP	20 (17.5%)
Personal contact	15 (13.2%)
The advice can also be discussed with the GP	7 (6.1%)
Extra stimulation by a GP is possible	6 (5.3%)

## Supplementary Materials

The following are available online at http://www.mdpi.com/1660-4601/15/7/1475/s1, File S1: Completed COREQ checklist.
