# Supplementary material for: Process Evaluation of an eHealth Intervention Implemented into General Practice: General Practitioners’ and Patients’ Views
Source: Int J Environ Res Public Health. 2018 Jul 12;15(7):1475. doi: 10.3390/ijerph15071475 (PMC6069123; doi:10.3390/ijerph15071475)
Supplement: Supplementary file 1 [file ijerph-15-01475-s001.pdf]

Consolidated criteria for reporting qualitative research (COREQ): a 32-item checklist for interviews and focus groups

Domain 1: Research team and reflexivity

Personal characteristics

1. Interviewer/facilitator. Which author/s conducted the interview or focus group?

Jolien Plaete (JP) and Janne Schepers (JS) conducted the interviews.

2. Credentials. What were the researcher's credentials? E.g. PhD, MD

JP: PhD candidate in Health Sciences
JS: PhD candidate in Pharmaceutical Sciences

3. Occupation. What was their occupation at the time of the study?

JP and JS were PhD students at the time of the study.

4. Gender. Was the researcher male or female?

JP and JS are both female reseachers.

5. Experience and training. What experience or training did the researcher have?

JP has a Master's degree in Health Education and Health Promotion
JS has a Master's degree in Drug Development (Pharmaceutical Sciences)

Relationship with participants

6. Relationship established. Was a relationship established prior to study commencement?

No relationship with the participants was established before the commencement of the study.

7. Participant knowledge of the interviewer. What did the participants know about the researcher? e.g. personal goals, reasons for doing the research

The participants knew that 'MyPlan 1.0' was created by Ghent University and conducted the study in order to explore its implementation in general practice.

8. Interviewer characteristics. What characteristics were reported about the interviewer/facilitator? e.g. Bias, assumptions, reasons and interests in the research topic

Specific characteristics of the researchers (e.g. training, profession) can always have an influence on data collection and analysis. Nevertheless, we created strict protocols to carry-out the interviews and to analyse the data to minimize bias.

Domain 2: study design

Theoretical framework

9. Methodological orientation and Theory. What methodological orientation was stated to underpin the study? e.g. grounded theory, discourse analysis, ethnography, phenomenology, content analysis

The interviews were thematically analysed using a combination of axial coding and inductive coding.

Participant selection

10. Sampling. How were participants selected? e.g. purposive, convenience, consecutive, snowball

The general practices were a convenience sample. Fifteen of the 19 GPs who implemented 'MyPlan 1.0' volunteered for the interviews.

11. Method of approach. How were participants approached? e.g. face-to-face, telephone, mail, email

The GPs were recruited via email messages, telephone calls and advertisements on association websites of GPs.

12. Sample size. How many participants were in the study?

Fifteen GPs were interviewed.

13. Non-participation. How many people refused to participate or dropped out? Reasons?

Four GPs refused to participate in the interviews.

Setting

14. Setting of data collection. Where was the data collected? e.g. home, clinic, workplace

The interviews took place at the general practice or via a telephone call. The interviews were audio-recorded with permission of the participants.

15. Presence of non-participants. Was anyone else present besides the participants and researchers?

There was no presence of non-participants.

16. Description of sample. What are the important characteristics of the sample? e.g. demographic data

The mean age was 47.2 (±12.2) years and the mean number of years of experience was 21.7 (±12.8) years. Eight (53%) were male, 9 GPs (60%) worked in group practices, and 6 worked solo (40%).

Data collection

17. Interview guide. Were questions, prompts, guides provided by the authors? Was it pilot tested?

All questions are provided in additional file 2.

18. Repeat interviews. Were repeat interviews carried out? If yes, how many?

There were no repeat interviews carried out.

19. Audio/visual recording. Did the research use audio or visual recording to collect the data?

All verbalizations were voice-recorded.

20. Field notes. Were field notes made during and/or after the interview or focus group?

No.

21. Duration. What was the duration of the interviews or focus group?

The average duration of an interview was 30 minutes.

22. Data saturation. Was data saturation discussed?

Disagreements were discussed by the two researchers until consensus was reached and interrater reliability was good (single measures ICC = 0.72). The final coding scheme was used to code all transcripts. Finally, all codes of the transcripts were mutually compared and interpreted.

23. Transcripts returned. Were transcripts returned to participants for comment and/or correction?

No.

Domain 3: analysis and findings

Data analysis

24. Number of data coders. How many data coders coded the data?

Two data coders (JP and NH) coded the data.

25. Description of the coding tree. Did authors provide a description of the coding tree?

Yes. See figure 2.

26. Derivation of themes. Were themes identified in advance or derived from the data?

The themes were identified in advance. There were five themes: (1) the delivery mode (i.e. use of the tablet and flyers), (2) the target group (i.e. was the intervention delivered to healthy patients and thus used for primary prevention), (3) barriers and facilitating factors for implementation, (4) discussion of the advice and action plan by GPs and (5) other ideas for future implementation.

27. Software. What software, if applicable, was used to manage the data?

The qualitative data analysis software nVivo 11 (QSR International Pty. Ltd. Version 11, 2015) was used to manage the data.

28. Participant checking. Did participants provide feedback on the findings?

No.

Reporting

29. Quotations presented. Were participant quotations presented to illustrate the themes / findings? Was each quotation identified? e.g. participant number

Yes.

30. Data and findings consistent. Was there consistency between the data presented and the findings?

Yes.

31. Clarity of major themes. Were major themes clearly presented in the findings?

Yes.

32. Clarity of minor themes. Is there a description of diverse cases or discussion of minor themes?

Yes.
